# Alterations of miR-16, miR-let-7a and their target genes expression in human blastocysts following vitrification and re-vitrification

**DOI:** 10.1186/s12958-021-00842-w

**Published:** 2021-10-09

**Authors:** Maryam Daneshvar, Mansoureh Movahedin, Mohammad Salehi, Mehrdad Noruzinia

**Affiliations:** 1 Department of Anatomical Sciences, Faculty of Medical Sciences, Tarbiat Modares University, Tehran, Iran; 2 Cellular and Molecular Biology Research Center, Shahid Beheshti University of Medical Sciences, Tehran, Iran; 3 Department of Medical Genetics, Faculty of Medical Sciences, Tarbiat Modares University, Tehran, Iran

**Keywords:** Human blastocyst, Re-vitrification, microRNA, Apoptosis, Implantation

## Abstract

Embryo cryopreservation is a widely used technique in infertility management and today is an essential part of assisted reproductive technology (ART). In some cases, re-vitrification can be applied to good quality supernumerary warmed embryos that have not been transferred in the present cycle. However, there is no study about re-vitrification impact on microRNA and gene expression in human embryos. The purpose of this study is to evaluate miR-16, miR-let7a and target genes expression in in vitro produced human blastocysts following re-vitrification.

Day3 embryos obtained from ICSI cycles of fertile couples referring for family balancing program were biopsied and cultured individually. On the fourth day (post-ICSI) male ones (choices of their parents) were transferred and the females (good quality embryos) were donated for research. Donated embryos were cultured to blastocyst stage and assigned to three groups: fresh, vitrified and re-vitrification. Embryos were vitrified on Cryotech carriers. Then blastocysts of three groups were individually assessed for expression of miR-16, miR-let7a and target genes.

The results showed that re-vitrification of human blastocysts did not affect the ability to re-expand in culture. In addition, significant decrease was observed in miR-16 and miR-let7a expression in re-vitrified group compared to fresh (*p* < 0.05). A significant upregulation of the target genes ITGβ3 and BCL-2 in re-vitrified and vitrified embryos was observed compared to the fresh group (*p* < 0.05). The expression of BAX as a pro-apoptotic gene showed a significant decrease in re-vitrification group comparing with the fresh one (*P* < 0.05).

The results of this research indicated that re-vitrification of embryos changes the expression of miR-16, miR-let-7a and their target genes. These alterations include increased expression of BCl-2 and ITGβ3 genes which play important roles in embryo survival and implantation, respectively. Clinical proof of these effects requires further research.

## Introduction

Embryo cryopreservation is one of the most widely used techniques in infertility management. This technique plays a key role in preserving fertility potential of those suffering from premature ovarian failure and also provides an embryo resource to be thawed/warmed for subsequent use in further treatment cycles. Furthermore, embryo “re-freezing” due to the embryologist’s discretion can be applied to good quality supernumerary thawed/warmed embryos that have not been transferred in the present cycle. Sometimes re-vitrfication becomes inevitable, such as in cases like vitrification of embryos whose oocytes of origin have been vitrified before or embryos genetically tested after warming and many of them are healthy and transferable yet supernumerary [1–3].

In 2001, Fahrat et al. reported the first successful pregnancy and birth from transfer of human embryos that had been subjected to two freeze-thaw cycles [4]. Few studies have been performed on re-verification of mouse embryos, indicating re-verification did not have any effects on developmental potential and gene expression [5, 6]. Re-verification of embryos seems to be a successful and useful method for preserving extra embryos, but so far, its safety and genetic and epigenetic effects of re-verification/warming of human embryos have not been studied.

MicroRNAs are innate, small noncoding RNAs with 20–23 nucleotides in length, having an important role in controlling posttranscriptional gene expression and are regulated by genetic and environmental conditions. To date the importance of microRNAs has been recognized in primary developmental steps including embryo implantation, development, cell growth, and differentiation in many species from C.elegans to mammals [7, 8].

Today it has been shown microRNAs play key roles during implantation [8]. MiR-let7 is among the first discovered microRNAs which is phylogenetically preserved as well. Previous studies indicate decreased levels of this microRNA in mouse embryos prior to implantation [9, 10] In a study by Liu et al. [11] it was shown that let-7a is involved in implantation process, partly via regulation of integrin-β3 expression. Integrins are transmembrane heterodimer glycoproteins and are expressed by trophectoderm during implantation and mediate embryonic adhesion through binding to an extracellular mediatory ligand [12].

Nowadays research has shown microRNAs are directly and indirectly involved in apoptosis process. Apoptosis or programmed cell death is an important factor in embryonic developmental arrest, which is triggered by activation, expression and regulation of some apoptotic gene families such as *BCL* and caspase, as well as microRNAs (miRNAs) [13, 14]. MicroRNA-16 has an effective regulatory role in several cellular and biological processes including apoptosis, cell cycle and inhibition of cell proliferation and differentiation. *BCL-2* is a target for microRNA-16 and its decline by microRNA-16 leads to apoptosis [15]. Chimino et al. [16] have shown miR-15 and miR-16 expression are inversely related to Bcl-2 expression in acute lymphatic leukemia. Zhao et al. [17] observed a higher expression of miR-16 in cryopreserved mouse blastocyst compared to the fresh ones.

Based on our knowledge in this field, there is no study concerning the effect of re-vitrification/re-warming and repeated exposure to cryoprotectants on human embryos. Therefore, in the present study expression of miR-let7a, miR-16 and their target genes will be studied in human blastocysts following vitrification and re-vitrification.

## Methods and material

### Ethical considerations

Blastocyst collection was done regarding ethical codes and approval of Medical Ethics Committee of Tarbiat Modares University, Iran (IR.MODARES.REC.1397.053). Human blastocysts were donated by healthy couples seeking sex-selection (male embryo favored) for family balancing in Gandhi Infertility Center (Tehran, I.R.I.), after signing a written informed consent. It is worth mentioning family balancing is only allowed for couples with at least two children of one sex (e.g. two girls), so sex selection is done for the third offspring.

### Blastocyst collection

About 30 good quality human blastocysts donated by fertile couples in reproductive ages (25–35 years old) from family balancing cycles in Gandhi Hospital Infertility Center were included in our study (15 good quality blastocysts for microRNA and gene expression analysis and 15 good quality blastocysts for TUNEL assays). Ovulation stimulation protocol was GnRH-Antagonist utilizing Cetrotide Antagonist (0.25 mg/day) (MerckSerono, Germany) in combination with Recombinant Gonadotropin SinalF (75 IU daily) (SinaClon, Tehran, Iran) and HMG Menogon (75 IU daily) (Ferring, Germany). Once the minimum of three follicles larger than 17 mm in diameter were visualized in the sonogram, 10,000 IU hCG (IBSA, Switzerland) was injected and after 36 h, oocyte retrieval was commenced under ultrasound guidance. Fertilization by ICSI were performed following standard protocols. All culture media were purchased from Life Global Company (Denmark). After injection, oocytes were cultured in Life Global medium until day 3. On the 3rd day, eight cell embryos were placed in Ca + 2 and Mg + 2 free culture medium to disrupt cell adhesion. Embryo biopsy was performed using Eppendorf Micromanipulators (Micromanipulator TransferMan, Eppendorf, Hamburg, Germany). To create an artificial hole in the zona pellucida, hatching was performed mechanically. A single blastomere was removed from each embryo and was assessed for the presence and number of Y and X chromosome. For sex determination of embryos, fluorescence in situ hybridization (FISH) technique carried out on biopsied blastomers After blastomere biopsy of three-day embryos they were cultured individually till day four (post-ICSI) and male ones (choices of their parents) were transferred and the females (good quality embryos) were donated to the research. Donated embryos were cultured to blastocyst stage and then evaluated under inverted microscope (Nikon, Japan) and sorted by Gardner’s criteria [18]. Gardner scoring system is based on the expansion state of the blastocyst and on the consistency of the inner cell mass (ICM) and trophectoderm cells. In this research we assessed fifth-day good quality (AA or AB: ICM with several tightly packed cells and trophectoderm with numerous cells forming a cohesive epithelium) blastocysts which were in developmental stages of “full blastocyst”, “expanding” and “hatching”. The inclusion and exclusion criteria of the collected embryos are given in Table 1.

**Table 1:** The inclusion and exclusion criteria of the collected embryos

Criteria	Inclusion Criteria	Exclusion Criteria
Fertility status	Fertile couple Healthy couples referring for family balancing	Infertile couple
Maternal age	25-35 yr	
Stimulation protocol	Controlled ovarian stimulation	High responder
Gender of the embryo	Female	Male
Chromosomal makeup of the embryo	No sex chromosome aneuploidies	sex chromosome aneuploidies
Morphology of the embryo (Gardner’s classification)	High grade blastocyst (AA, AB, BA)	Low grade blastocyst

### Study design

**Fig. 1:**
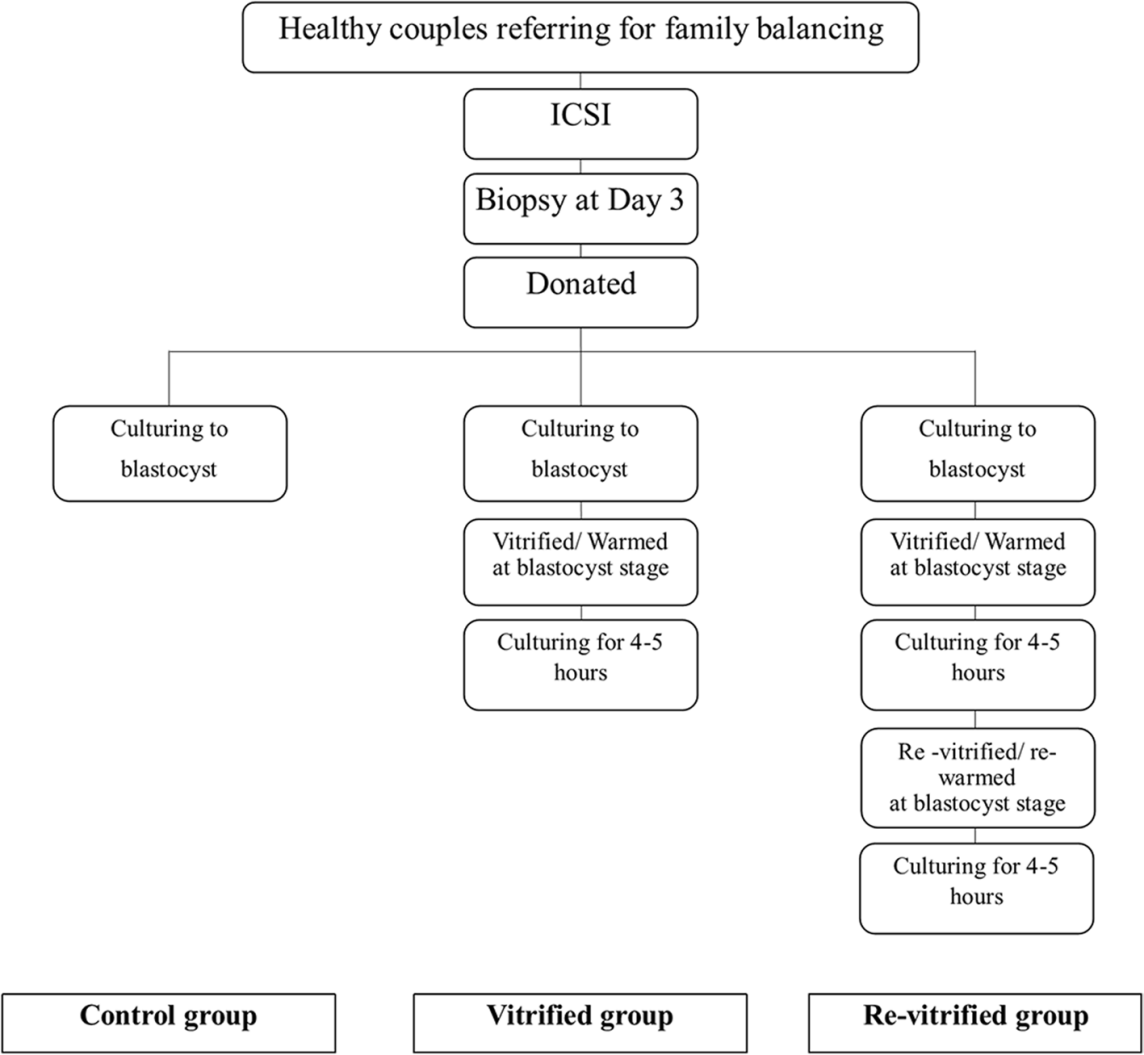
Schematic presentation of the study design. Abbreviations, ICSI: intra cytoplasmic sperm injection

Study design is illustrated in Fig. 1. As shown in the figure blastocysts were randomly assigned to three groups: fresh, cryopreserved once (vitrified) and cryopreserved twice (re-vitrification).

1. Fresh group: In fresh group, blastocysts were cultured in Life Global medium on the fifth day around 8–10 h, then assessed directly.
2. Vitrification group: in vitrification group blastocysts were vitrified and then warmed and cultured for 4 h post-warming in LifeGlobal medium and good-quality embryos were studied.
3. Re-vitrification group: In the third group blastocysts were vitrified, warmed, cultured (4 h) and re-vitrified, warmed, cultured (4 h) and then assessed if they had survived with good quality.

It is noteworthy that in our study embryos were assessed individually. Since the gender of preference for transfer was male by donating couples, female embryos were chosen for this research. Five blastocysts were evaluated in all experimental groups.

### Vitrification and warming procedures

Blastocysts were first exposed to the equilibration solution containing 7.5% (v/v) Ethylene Glycol (EG, Sigma Aldrich Chemical Company, Germany), 7.5% (v/v) Di methyl sulfoxide (DMSO, Sigma Aldrich Chemical Company, Germany) in HTF medium (Genocell, Tehran, Iran) supplemented with 20% HSA (Human serum albumin, Switzerland) for 15–20 min at room temperature, and then were placed in the vitrification medium 15% EG, 15% DMSO and 0.5 M sucrose (Merck, Germany) in HTF medium plus 20% HSA for up to 1 min. The embryos were loaded on cryotech carriers (Tokyo, Japan) with minimum medium and immediately plunged into liquid nitrogen (LN2). For warming, cryotech was removed from LN2, immediately placed in warming medium (1.0 M sucrose in HTF medium plus 20% HSA) and incubated for 1 min at 37 C. Blastocysts were then placed in dilution solution 1 and 2 (DS1:0.5 M sucrose in HTF plus 20% HAS; DS2: 0.25 M sucrose in HTF plus 20% HAS) each for 3 min. After washing in HTF plus 20% HSA, the blastocysts were cultured for 4 h in LifeGlobal medium at 37 °C in 6% CO_2_ in an incubator atmosphere.

### Evaluation of warmed blastocysts survival

Re-expansion of blastocysts was evaluated by an inverted microscope (Nikon, Japan) after warming and during 2-4 h of culture. The literature has shown re-expansion ability of the blastocyst in the first few hours after warming as an indicator of viability, and high-quality embryos possess faster re-expansion ability after warming. Embryos with poor quality after warming were excluded from the study.

### Simultaneous RNA extraction and cDNA synthesis

In this phase of study, 15 blastocysts were randomly divided in three groups. Blastocysts were washed individually in droplets of PBS-0.1% PVA (phosphate-buffered saline/Polyvinyl alcohol) and then transferred into microtubes containing 2 μL of lysis buffer [19]. After placing the microtubes on ice, 3 μL Random Hexamer, 5 μL Nuclease-free water, 2 μL miR-16 RT primer, 2 μL miR-let 7a RT primer and 2 μL snord 234 RT primer were added and placed in Thermocycler for 5 min at 75 °C. Then microtubes were placed on ice and for cDNA synthesis 10 U Rnase inhibitor, 10 μL dNTP, 200 U RT enzyme and 5 × RT buffer were added and remained in thermocycler (applied Biosystems) at 25 °C for 10 min, 37 °C for 15 min, 42 °C for 45 min, and 72 °C for 10 min. After the end of reverse transcription, resulting cDNAs were kept at 4 °C overnight. To make sure cDNAs exist, RT-PCR was proceeded for each microtube by adding 5 μL master mix (Taq DNA polymerase master mix red, Ampliqon, Denmark), 1 μL cDNA, 3 μL nuclease-free water and 1 μL b2m primer with the thermal order of 94 °C for 4 min (denaturation), 94 °C for 30s (denaturation), 60 °C for 30s (annealing), and 72 °C for 45 s (extension), followed by 40 cycles. A final elongation step was done at 72 °C for 10 min. Finally, PCR products were run on 2% Agarose gel and visualized by short-wave UV. Complementary DNA synthesized using cDNA Synthesis Kit (Yekt Tajhiz, Iran).

### Reverse transcription–qPCR analysis

In this study expression of *BAX*, *BCL2*, *ITGβ3*, miR-let7and miR-16 was assessed by RT-qPCR technique using RealQ Plus Master Mix Green, amplicon (Denmark) and in Applied Biosystems Step One Plus Real-time PCR instrument. The primer sequences which are used for RT-qPCR, are given in Table 2. The final volume of each reaction was 13 μL including 7 mmol/L Master mix, 4 mmol/L nuclease-free water, 1 mmol/L mixture of forward and reverse primers of each gene and microRNA and 1 mmol/L of the synthesized cDNA. The temporal program of RT-qPCR was alternatively adjusted in 95 °C for 2 min, in 95 °C for 5 s for denaturation, in 60 °C for 30s, in 72 °C for 10s for amplification, with 40 cycles of extension. All of the samples were run in duplicate and each of the experiments was conducted in triplicate. B2m reference gene was used as a normalizer for target gene expression and snord was used as a normalizer for microRNA expression.

**Table 2:** Primers used for quantitative gene expression

Gene name	Primer sequence
ITGβ3	F: AGTAACCTGCGGATTGGCTTC
	R: GTCACCTGGTCAGTTAGCGT
BCL-2 (*Homo sapiens* BCL2 apoptosis regulator (BCL2), transcript variant alpha)	F: GAGAAATCAAACAGAGGCCG
	R: CTGAGTACCTGAACCGGCA
BAX	F: CCCGAGAGGTCTTTTTCCGAG
	R: CCAGCCCATGATGGTTCTGAT
B2M	F: GACTGGTCTTTCTATCTCTTGTAC
	R: ATGTCTCGATCCCACTTAACTATC
miR-16	F: GTATACTAGCAGCACGTAAAT
	R: GTGCAGGGTCCGAGGT
miR-let-7a	F: GTATACTGAGGTAGTAGGTTG
	R: GTGCAGGGTCCGAGGT
SNORD	F:AGATTTAACAAAAATTCGTCAC
	R:GAGCAGGGTCCGAGGT

### TUNEL assay and Hoechst staining

Fifteen blastocysts were used for TUNEL assay. TUNEL assessment was executed using an in situ cell death detection kit TMR red (Roche, Mannheim, Germany) based on the company’s instructions. After washing the blastocysts in droplets of PBS-0.1% PVA (phosphate-buffered saline/ Polyvinyl alcohol), they were fixed in 4% of paraformaldehyde in PBS for 1 h at room temperature. Then, the blastocysts were permeabilized in 0.1% triton-× 100 + 0.1% sodium citrate for 2 min and incubated with TUNEL reaction mixture for 1 h at 37 °C in the dark. For positive control of TUNEL assay, fixed and permeabilized blastocysts were incubated with 50 IU/ml of DNase I enzyme (Promega, Germany) for 20 min at 37 °C before TUNEL labelling. On the other hand, for Negative controlling, the embryos were incubated with TUNEL reagent without DNase treatment. After TUNEL, the blastocysts were washed in PBS-0.1% PVA and all of the blastocysts’ nuclei were stained using Hoechst33342 (10 μg/ml) for 30s at room temperature in the dark. The blastocysts were three times washed and mounted on the glass slides. Finally, all number of nuclei and apoptotic nuclei were obtained based on the optical image of whole-mount blastocysts using fluorescence microscope (Nikon, Japan).

### Statistical analysis

The SPSS program (ve.16) was used for statistical analysis of the data. Analysis of molecular results was carried out using REST 2009 program (Qiagen). The Kolmograph-Smirnov Statistical test results showed normal distribution of data. One-way analysis of variance (ANOVA) followed by Tukey’s HSD post-hoc was performed for evaluating the significance of the qPCR and TUNEL results. The data reports were considered as means± SD and the statistical significance was obtained based on *P* < 0.05.

## Results

Thirty donated high-quality human embryos from mothers between 25 and 35 years of age were assessed individually in this study. The embryos were cultured to blastocyst stage and then underwent vitrification and warming. The survival rate of blastocysts was 100% following vitrification/warming or re-vitrification/re-warming in both groups.

The expression of miR-16 and miR-let7 and their target genes *ITGβ3*, *BCL2* and *BAX* was assessed based on the experimental results.

**Fig. 2:**
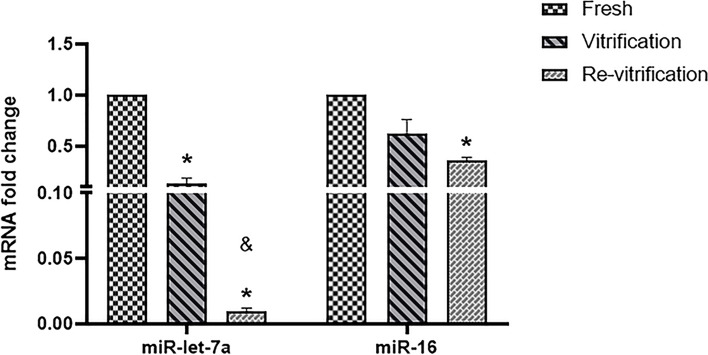
The Bar graph showing the effects of vitrification and re-vitrification on the expression level of the microRNAs in human blastocysts (means±SD). * indicates *P* < 0.05, between fresh and vitrification groups; also, fresh and re-vitrification groups & indicates *P* < 0.05, between vitrification and re-vitrification groups

There was significant decrease in miR-let7a expression in vitrified group compared to the fresh and for re-vitrified versus fresh group (*p* < 0.05). Also, miR-let7a expression showed a significant decrease in re-vitrified group compared to vitrified (*P* < 0.05) (Fig. 2).

The expression of miR-16 showed a significant decrease in re-vitrified group versus fresh group (Fig. 2). However, miR-16 expression did not show a significant difference in vitrified group compared to the fresh and in re-vitrified group compared to vitrified (*P* > 0.05). (Fig. 2).

**Fig. 3:**
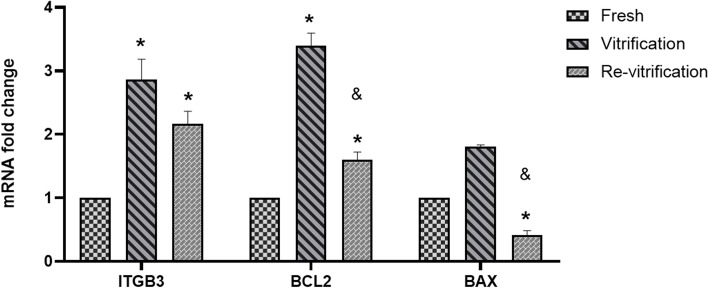
The Bar graph showing the effects of vitrification and re-vitrification on the expression level of the *ITGβ3*, *BCL2* and *BAX* in human blastocysts (means±SD). * indicates *P* < 0.05, between fresh and vitrification groups; also, fresh and re-vitrification groups. & indicates *P* < 0.05, between vitrification and re-vitrification groups

Expression of *ITGβ3* gene as a miR-let7a target gene that is involved in implantation, showed a significant increase in vitrified embryos compared to fresh embryos and re-vitrified embryos compared to that of the fresh ones. However, *ITGβ3* gene expression did not show a significant difference in re-vitrified group compared to vitrified group (*P* < 0.05) (Fig. 3).

There was significant increase in anti-apoptotic *BCL2* expression (*Homo sapiens* BCL2 apoptosis regulator) as a target gene of miR-16 in vitrified group compared to fresh and in re-vitrified group compared to fresh (*P* < 0.05) (Fig. 3).

The expression of *BAX* as a pro-apoptotic gene showed a significant decrease in re-vitrified group compared to the fresh one (*P* < 0.05) but there was no significant change in the vitrified group compared to the fresh (*P* > 0.05) (Fig. 3).

**Fig. 4:**
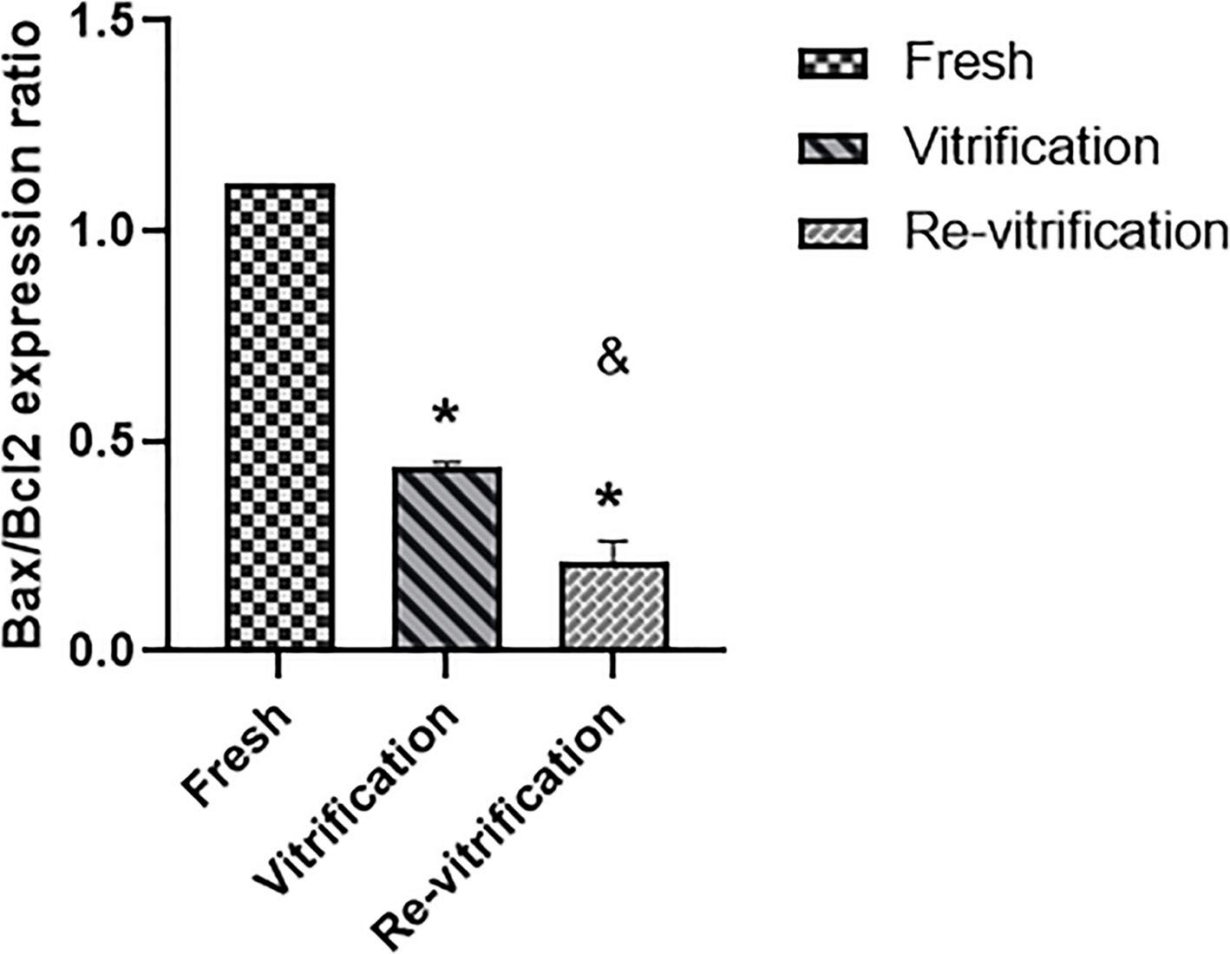
The Bar graph showing the effects of vitrification and re-vitrification on *BCL2*/*BAX* expression ratio (means±SD). * indicates *P* < 0.05, between fresh and vitrification groups; also, fresh and re-vitrification groups. & indicates *P* < 0.05, between vitrification and re-vitrification groups

The ratio of *BAX/BCL-2* expression was calculated in the fresh (1.11), vitrified (0.44) and re-vitrified groups (0.21). There was significant decrease in *BAX/BCL-2* expression ratio in vitrified group compared to the fresh and in re-vitrified group compared to the fresh (*P* < 0.05) (Fig. 4).

**Fig. 5:**
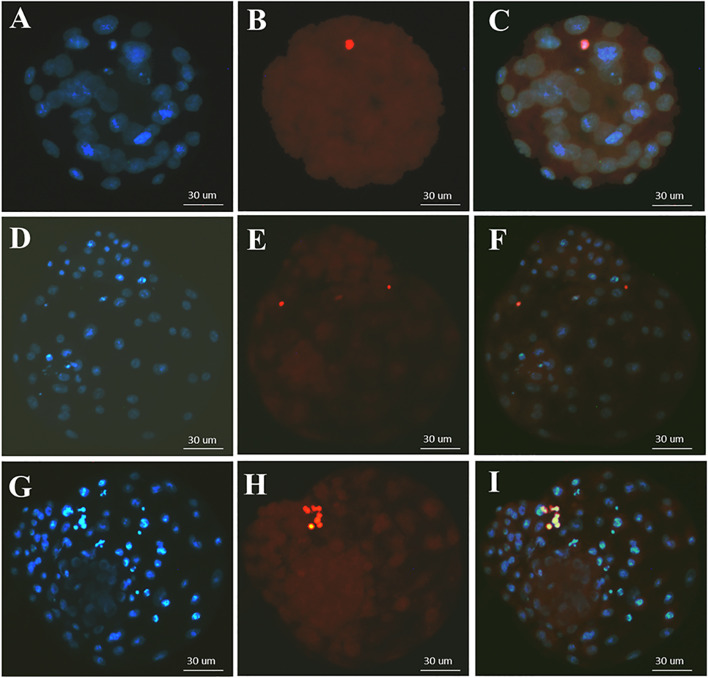
TUNEL staining in human blastocyst. The nuclei are stained by hoechst (blue color). the apoptotic nuclei are stained by TUNEL (red color). **A-C**, Apoptosis in fresh human blastocysts. **D-F**, Apoptosis in vitrified/warmed human blastocysts. **G-I**, Apoptosis in re-vitrified/re-warmed human blastocysts

**Fig. 6:**
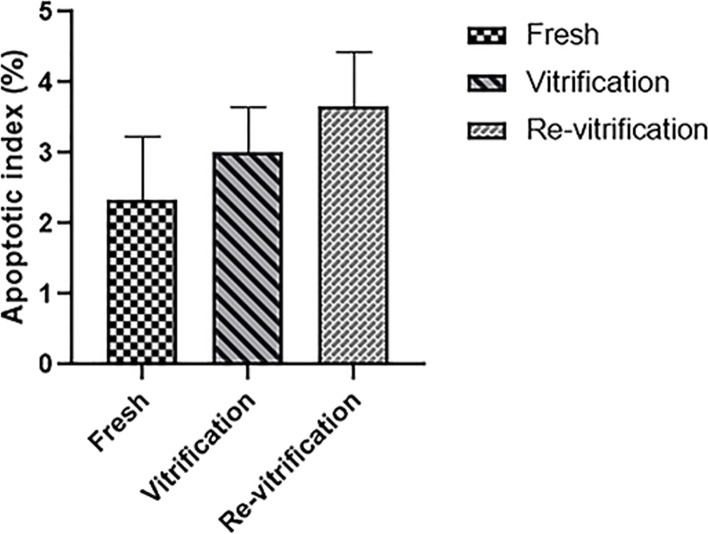
Apoptotic index in human blastocycst following vitrification and re-vitrification (*n* = 5) (means±SD)

**Fig. 7:**
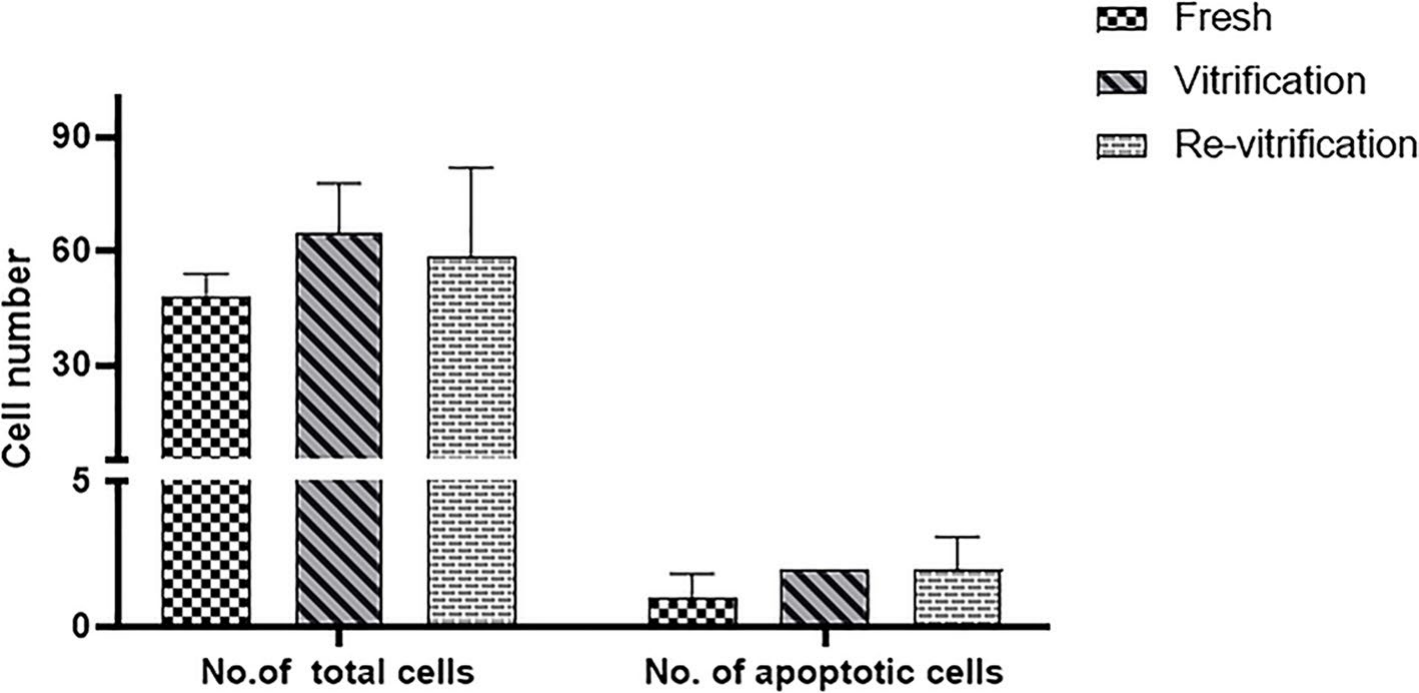
The average number of total cells and total apoptotic cell in blastocysts of each group (means±SD)

The TUNEL assay was conducted on human blastocysts to evaluate apoptosis (Fig. 5). There was no significant difference in apoptotic index in vitrification and re-vitrification groups compared to their fresh counterparts (Fig. 6). Also, the average number of total cells and total apoptotic cell in blastocysts of each group is shown in Fig. 7.

## Discussion

The current study evaluated the effect of vitrification and re-vitrification on survival of blastocyst. These results showed vitrification and re-vitrification did not have any effect on re-expansion capability of blastocysts after warming. In agreement with current results, other studies have repeatedly shown that vitrification and re-vitrification do not have any effect on viability of blastocysts and they are capable of progressing to successful pregnancies and healthy live births [5, 20, 21].

Also, we evaluated the effects of vitrification and re-vitrification on alterations of microRNA expressions involved in apoptosis (miR-16), implantation (miR-let7a) and target genes (*BCL2*, *BAX*, *ITGβ3*) in human blastocysts.

MicroRNAs play an important functional role in embryo developmental capabilities such as implantation and differentiation [7, 8]. Recently, many studies have been done on effects of Assisted Reproductive Technology on microRNA expression levels in gametes and embryo. These studies showed that manipulation of embryos or gametes causes alterations in microRNA expression [22–24]. In a study by Azizi et al. in 2019 on mouse embryos it was shown that let-7a expression significantly decreased in IVF-driven blastocysts versus in vivo ones [25]. Zhao et al. showed vitrification of mouse blastocyst resulted in significant alterations of microRNA transcriptome, which may affect implantation potential of vitrified blastocysts [17].

Our findings indicated significant decrease of miR-let7a expression and, subsequently, significant increase of *ITGβ3* gene expression in vitrified embryos compared to the fresh group. Furthermore, we observed a significant decrease in mir-let7a and an increase of *ITGβ3* expression in re-vitrified embryos compared to the fresh ones.

MiR-let7a belongs to Lethal 7 family which plays a critical role in murine and human blastocyst implantation through regulating the expression of integrin Beta3, *Vav3* and Dicer genes. Recent reports have indicated that integrin Beta3, *Vav3* and Dicer induce trophoblastic activities in order to implant successfully [26]. In 2012 Liu et al. reported expression of miRNA let-7 decreases considerably in embryos ready to implant and on the other hand, decreased expression of miRNA can have an important effect on increasing integrin beta expression, which itself plays a key role in embryo adhesion [11]. Integrins are heterodimeric transmembrane glycoproteins which are expressed by the trophectoderm at the implantation stage and mediate embryonic adhesion via attaching to an extracellular mediating ligand [27, 28].

A report by Heydari et al. in [9] described that mir-let7a had a significant decrease while *ITGβ3* gene expression was significantly increased in vitrified mouse blastocysts comparing with the fresh group. The present results and the above-mentioned studies indicate that Assisted Reproductive Technology has an effect on microRNA expression, however the accurate impact of this alteration on implantation is not clear.

Numerous investigations have reported higher implantation and pregnancy rates in vitrified embryos comparing with the fresh ones [29, 30]. This improvement seems to be mostly due to better embryo/endometrium synchronization and absence of adverse effects of controlled ovarian hyperstimulation (COH) [31] but whether decreased downregulation of miR-let7a and subsequently upregulation of *ITGβ3* following vitrification contributes to these clinical outcomes is unknown and requires further research.

Also, effects of re-vitrification on expression of miR-16, *BCL2* and *BAX* were assessed in our study. miR-16 is situated on chromosomal position of 13q14 and is highly conserved in mammalian species which testifies to its paramount value in physiological processes and normal development [32]. Studies have indicated that *BCL2* is a target gene of miR-16 and this microRNA induces apoptosis through inhibiting *BCL2* [9, 16]. *BCL2* protein as a proto-oncogene is involved in intracellular apoptosis regulation and generally plays a crucial role in inhibiting cell apoptosis and early embryo development [33].

In this research, a significant decrease in the expression of miR-16 in vitrified and re-vitrified groups was observed compared to that of fresh. Also, the level of *BCL2* expression which is considered as one of miR-16 targets, had significant increase in both vitrified and re-vitrified groups compared to fresh. In this study *BAX* expression in re-vitrified embryos had a significant decrease compared to fresh ones but we did not observe a significant decrease compared to the vitrified ones. Upregulation of anti-apoptotic *BCL2* expression and down-regulation of proapoptotic *BAX* expression, resulted in a reduction of the *BAX*/*BCL2* ratio in both vitrified and re-vitrified groups compared to the fresh groups.

TUNEL is a method which has been widely used to detect apoptotic DNA fragmentation in individual cells. We used this technique to evaluate apoptotic cells in blastocysts. The apoptotic index obtained from the tunnel assay is 2.36, 3.02 and 3.61 in the control, vitrification and re-vitrification groups alternatively which are small and negligible values. Although there was a slight increase in the apoptotic index in re-vitrification group compared to vitrification group, however this slight increase was not significant and may be due to the small number of samples.

Similar results were also showed in a study by Heydari et al. in 2019 who reported that the vitrification of mouse embryos results in significant decrease of miR-16 expression and increase of *Bcl2* [9]. In another study by Turathm et al. [34] it was shown that *Bcl2* gains higher expressions in vitrified immature canine oocytes compared to control. Contrarily, Majidi et al. reported that *Bcl2* displays a greater decrease in the re-vitrified mouse embryos compared to fresh and expression of *BAX* gene was up-regulated in the re-vitrified group [6].

Dahli et al. reported interesting results about effect of vitrification on *Bcl2* expression in two separate studies. In 2007 they observed significant downregulation of *Bcl2* and p53 in mouse zygotes following droplet vitrification method [35]. But in another study (2009), following modified droplet vitrification they reported significant upregulation of these genes in mouse zygotes following vitrification [36].

.It seems different pattern of gene expression observed in our research and other studies may be due to quality and developmental ability of the assessed embryos and also how vitrification and warming of these embryos was done.

In this study we assessed high-quality embryos donated by healthy fertile couples while embryos of infertile couples may respond differently to stress due to their deficiencies such as alteration of gene expression or DNA fragmentation and may end up proceeding to cell death. Yang et al. in [37] reported a relationship between *Bcl2* expression and quality of embryos and oocytes. They showed up-regulation of *Bcl2* in good-quality bovine embryos and oocytes compared to degenerated embryos. Numerous investigations have reported relationships between differential expression of genes and microRNA in patients with different spermatogenic impairments [38–40].

Previous studies have shown cell response to stress depending on the cell type, stress intensity and duration of stress exposure can be an initializer of either survival or cell demise [41, 42]. Increased levels of *BCL2* expression is a cell response to stress which acts through inhibiting cytochrome C from the mitochondrion [41]. Therefore, regarding previous reports, it seems high-quality embryos show a better adaptability and resistance toward stress, a response which can include increased expression of anti-apoptotic proteins and genes involved in implantation.

Also, the results in this study showed that the expression of *Bcl2* and *BAX* genes in the re-vitrification group was significantly reduced compared to the vitrification group and the expression of *ITGβ3* gene in the re-vitrification group was reduced compared to the vitrification group, however this decrease was not significant. In 2017, Yu et al. reported multiple cycles of freeze–thawing reduced RNA integrity in lung cancer tissues [43]. In another study in 2021, Kellman et al. showed that multiple freeze-thaw reduced the stability of poly A sequences in RNA [44] . The results of this study also showed a decrease in RNA following re-vitrification compared to vitrification group, which can indicate the effect of repeated freeze-thaw cycles on the RNA integrity.

It is important to be noted that the mentioned results in this section should be interpreted with caution and a number of limitations must be considered too as following:

1. Small number of samples examined due to difficult access to human blastocysts donated by fertile couples.
2. In this study the embryos were assessed individually, therefor due to the small volume of extracted RNA from any blastocyst, it was not possible to evaluate the expression of further
3. Evaluation of high-quality embryos donated by healthy fertile couples while embryos of infertile couples may respond differently.

## Conclusion

Post-cryopreservation survival of human blastocysts is potentially associated with the *miR-16* and miR-let-7a which are related to regulation of implantation and apoptosis processes, respectively. The results of this study showed that vitirification and re-vitirification did not have a negative effect on the expression of genes involved in implantation and apoptosis, which seems to be due to the adaptability to stressful conditions of quality embryos obtained from healthy and fertile couples. On the other hand, gene expression in the re-vitirification group decreased compared to the vitirification group, which may indicate the effect of multiple freeze-thaw cycles on RNA integrity. However, the exact effect of re-vitirification on RNA integrity as well as gene expression requires further studies. Also, further clinical investigations are still required to assess responses of embryos belonging to sub-fertile/infertile couples that they may have less resistance potential to stressful situations.

## Data Availability

We declare that the data availability which support the findings of this study is available from the corresponding author in upon a reasonable request.
